# Environmental variables influencing tick anaphylaxis presentations: An observational study

**DOI:** 10.5415/apallergy.0000000000000222

**Published:** 2025-10-06

**Authors:** Katherine Duong, Melanie Burk, David Boettiger, Bronte Cross, Ayesha Owen, Jordan Symons, Andrew Ratchford, Sheryl van Nunen

**Affiliations:** 1 Northern Beaches Clinical School, Macquarie University, Sydney, Australia; 2 TiARA (Tick-induced Allergies Research and Awareness), Australia; 3 Epidemiology, Hunter New England Local Health District, New South Wales, Australia; 4 Kirby Institute, University of New South Wales, Sydney, Australia; 5 Northern Beaches Hospital, Sydney, Australia; 6 National Allergy Centre of Excellence, Australia; 7 The University of Sydney, Sydney, Australia

**Keywords:** Anaphylaxis, bushfires, humidity, rainfall, temperature, ticks

## Abstract

**Background:**

*Ixodes holocyclus*, the predominant tick species on the eastern coast of Australia, is responsible for the increasing incidence of tick anaphylaxis (TA) presentations. While ticks thrive under warm and humid conditions, the influence of environmental conditions on TA incidence remains unclear.

**Objective:**

This study aimed to correlate environmental variables with TA presentations in New South Wales (NSW), Australia, to predict tick seasons and forewarn residents of tick-endemic regions.

**Methods:**

Monthly temperature, rainfall, dewpoint, and fire extent data were collected from open-access government resources for Northern and Southern Coastal NSW, and Greater Sydney regions. These were compared with tick bite locations and dates from 278 TA patients from 2005 to 2022, using Pearson’s correlation.

**Results:**

In Greater Sydney, TA cases were weakly correlated with rainfall (r = −0.15), temperature (r = 0.26), and dewpoint (9 am: r = −0.22; 3 pm: r = −0.22), and a strong negative correlation with percentage area burned (r = −0.94). Northern and Southern coastal NSW TA cases exhibited similarly weak correlations with rainfall, temperature, dewpoint, and percentage area burned.

**Conclusion:**

Larger bushfires may reduce TA presentations through tick habitat destruction. However, the role of weather variables remains inconclusive, highlighting the need for further research to better understand tick ecosystems, which may aid public health initiatives for tick awareness.

## 1. Introduction

Worldwide, various infectious diseases have been associated with tick bites, such as borreliosis, also called “Lyme Disease,” tick-borne encephalitis, and Crimean-Congo hemorrhagic fever, reported across North America, Europe, and Asia [1]. In Australia, tick bites are known to cause allergic reactions, paralysis, and tick-borne infections, including Australian tick typhus, and rarely, Q fever or babesiosis [2–4]. Tick bites are becoming increasingly more common in Australia, and with this, an exponential rise in cases of tick-induced allergies, mammalian meat allergy following tick bite, and tick anaphylaxis (TA) has been observed [5]. Several fatalities have been reported as a consequence of tick anaphylaxis [6], and with mammalian meat allergy, potential for severe or fatal allergic reactions to meat, animal products, medications, and therapeutic goods containing alpha-gal arises [7–9].

In Australia, most tick bites come from the Australian paralysis tick (*Ixodes holocyclus*) [5], to which this study will largely refer. Presently, this species of tick is distributed along almost the entire eastern seaboard of Australia; however, it is predicted that ongoing climate change will expand their range, especially to the colder southern regions [10, 11]. Any life stage of *Ix. holocyclus* can be present throughout the year; however, the larval form will typically predominate in autumn, nymphs in winter, and adults in spring [12]. *Ix. holocyclus* tends to be more abundant from September to March, when conditions are favorably warmer and more humid, allowing eggs to hatch readily [13, 14]. Previous studies in Europe and Asia have explored the relationship between tick bites from other species and weather conditions, suggesting that warmer temperatures are associated with larger and more widespread tick populations and increased tick activity [15–17]. However, to date, there has been no research examining the impact of warmer and more humid weather conditions or bushfires on tick-induced allergies, including anaphylaxis. If indeed warmer weather predicts increased tick abundance and activity, the advent of climate change and increasing global temperatures warrants the need for greater tick bite precautions.

Establishing a correlation between environmental variables and TA cases may provide further insight and direction for tick-related public health initiatives. This may include alerting residents in tick-prone areas to specific seasons/environmental conditions that may observe higher than usual tick bite rates and when they must be more vigilant with tick bite prevention strategies. Furthermore, forecasts about environmental conditions may aid resource allocation in emergency departments (EDs) or urgent care centers, to be better equipped for presentations of tick allergies, possibly reducing tick bite-related morbidity and mortality in the long term. This study, therefore, aims to investigate the rates of TA presentations in the last 2 decades in relation to bushfire extent and weather variables in coastal New South Wales (NSW).

## 2. Methods

This study examined the relationship between patient demographic data and environmental variables.

### 2.1. Participant data

Patients who presented with TA and were managed in EDs in NSW, particularly in the Northern Beaches area, and other tick-endemic areas of coastal NSW and south-east Queensland, were referred to and reviewed by a consultant immunologist and allergist between 2005 and 2022. In this study, TA was clinically diagnosed when patients presented with symptoms consistent with anaphylaxis, such as respiratory compromise, shock, abdominal pain and vomiting, pruritus and urticaria, and perioral angioedema, following a tick bite, with no other obvious precipitants.

Patient information was obtained from electronic medical records. Information collected included patient address, date of tick bite/s, life stage of the tick when bitten, location of tick bite/s on body, reaction to the tick bite/s, and personal or family history of atopy. Patients who resided outside of NSW were excluded as there were too few patients to represent their areas. Patients without a recorded postcode or month and year of tick bite were also excluded, as these patients’ data were considered incomplete for analysis.

Human Research Ethics Committee approval was granted for this study (2022/NBH/025).

### 2.2. Environmental data

Open-access data from weather stations in local government areas (LGA) in the North Coast, Hunter, Central Coast, Metropolitan Sydney, Illawarra and Southern NSW regions on monthly rainfall, mean maximum temperature, and 9 am and 3 pm dewpoint temperatures from 2002 to 2022 was collected from the Australian Bureau of Meteorology website (Appendix 1; https://links.lww.com/PA9/A68). Weather data was then entered into a Microsoft Excel spreadsheet, averaged for larger NSW regions, and subsequently grouped into Northern NSW (includes North Coast, Hunter, and Central Coast), Greater Sydney, and Southern NSW (includes Illawarra and Southern NSW).

Open-access fire extent data (FESM-SEED) from the years 2016–2023 were obtained from the NSW Government Department of Planning and Environment. Percentage local land service area burned (% area burned) for Greater Sydney, Hunter, North Coast, and Southeast was used from these databases.

### 2.3. Data analysis

Averages of rainfall and temperature were taken from each month from 2005 to 2021, and averages of 9 am and 3 pm dewpoint temperatures were taken from each month from 2005 to 2010. The total numbers of TA cases per month were collated for the years 2005–2021 and divided into 3 corresponding regions (Northern NSW, Greater Sydney, and Southern NSW) based on the address patients provided. The number of cases was summated rather than averaged, as there were several months during the study period where cases were recorded to be zero. The year range restriction to environmental and patient data was applied due to large numbers of missing data points in some years. Monthly regional weather data were plotted against the number of TA cases in that region in the same month and analyzed using Pearson’s correlation. Similarly, % area burned in a region for each year was plotted against the number of TA cases in the same region/year and analyzed using Pearson’s correlation. A *P* value of less than 0.05 was considered statistically significant.

## 3. Results

**Figure 1.**
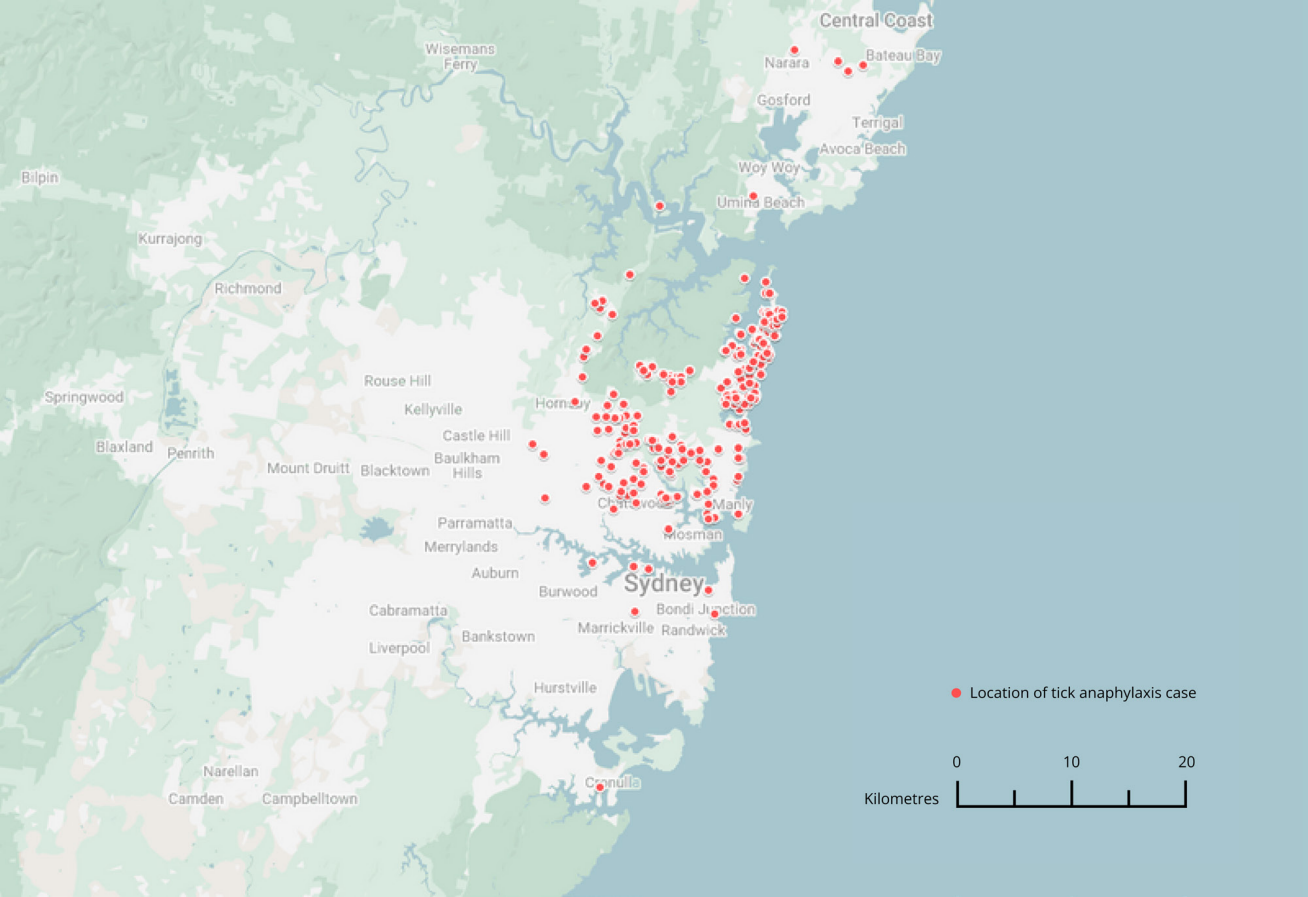
Geographical distribution of tick anaphylaxis cases within Greater Sydney (constructed using Google MyMaps). Appendix 3 shows a map of the entire study region https://links.lww.com/PA9/A70.

A total of 377 patients were reviewed. After applying the relevant exclusion criteria, data from 278 patients were included for analysis. Of these patients, 257 were located in Greater Sydney, 15 in Northern Coastal NSW, and 6 in Southern Coastal NSW. TA cases were concentrated in the Northern Beaches region of Greater Sydney (Figure 1). Patient demographics and presentations are summarized in **Table 1**, note that some patients were bitten by multiple ticks in different body locations. Patients presented to ED with typical anaphylaxis symptoms, including perioral angioedema, urticaria and pruritus, airway swelling and difficulty breathing, nausea and vomiting, dizziness or loss of consciousness. They were frequently treated with adrenaline, corticosteroids, and antihistamines, depending on the severity of their symptoms.

**Table 1.** Patient demographics and details of TA presentation

Age	
0–10	65
11–20	31
21–30	12
31–40	12
41–50	33
51–60	36
61–70	54
71–80	28
81+	7
Sex	
Female	135
Male	138
Not specified	6
Personal or family history of atopy	
Asthma	139
Eczema	106
Allergic rhinitis	145
Food allergy	35
Drug allergy	88
Insect allergy	58
Pet animal allergy	18
Location of tick bite on body	
Head	142
Neck	26
Chest	9
Back	14
Abdomen/pelvis	6
Upper limb	10
Lower limb	3
Not known	76
Life stage of tick	
Larval	1
Nymph	18
Adult	206
Not known	72

**Figure 2.**
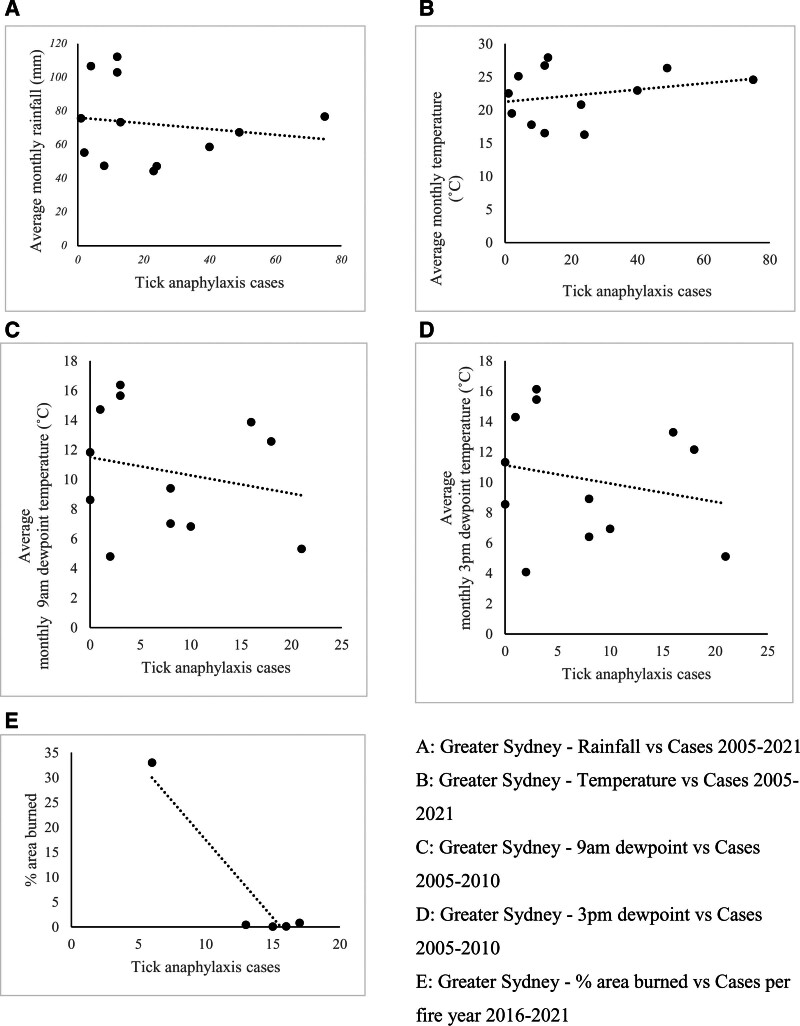
Scatter plots showing the correlation between weather data and tick anaphylaxis cases in Greater Sydney.

**Figure 3.**
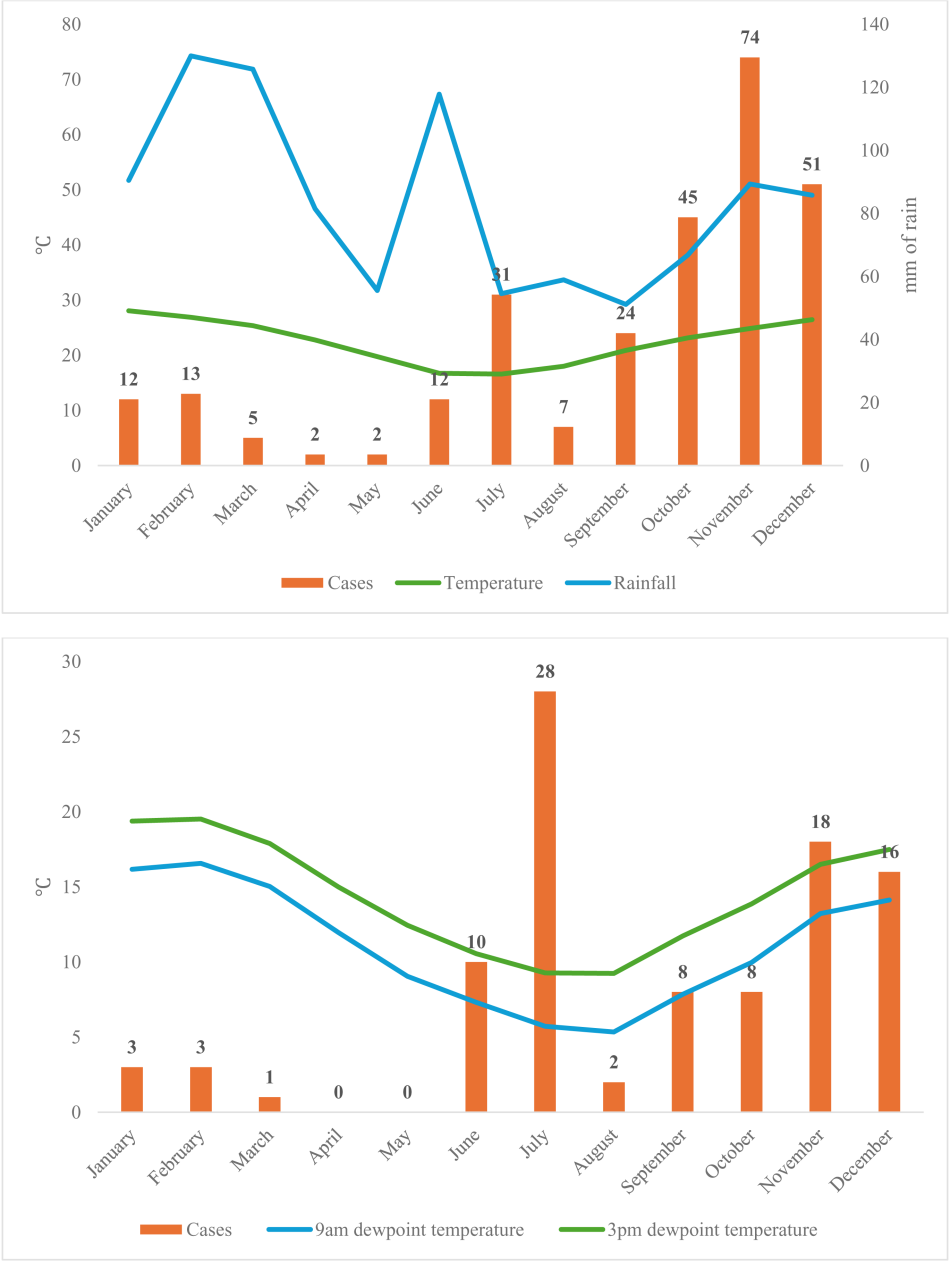
Top: Monthly TA cases in coastal NSW regions (Greater Sydney, Northern NSW, and Southern NSW) in 2005–2021 vs monthly average rainfall and temperature. Middle: Monthly TA cases in coastal NSW regions in 2005–2010 vs monthly average 9 am and 3 pm dewpoint temperatures. NSW, New South Wales; TA, tick anaphylaxis.

In Greater Sydney between 2005 and 2021, the number of TA cases were weakly correlated with rainfall (r = −0.15; *P* > 0.05), temperature (r = 0.26; *P* > 0.05) and between 2005 and 2010, dewpoint (9 am: r = −0.22; 3 pm: r = −0.22; *P* > 0.05), while there was a strong negative correlation with % area burned from 2016 to 2021 (r = −0.94; *P* < 0.05). Between 2005 and 2021, both Northern and Southern coastal NSW TA cases showed similarly weak correlations with rainfall (r = −0.46, −0.20 respectively; *P* > 0.05), temperature (r = −0.20, −0.27; *P* > 0.05), from 2005 to 2010, 9 am dewpoint (both r = −0.42; *P* > 0.05) and 3 pm dewpoint (r = −0.46, −0.41; *P* > 0.05), and weak correlations with % area burned (r = −0.40, 0.25, *P* > 0.05) in 2016–2021. These findings are summarized in Table 2, with plots of Greater Sydney data shown in Figure 2 and Northern and Southern Coastal regions shown in Appendix 2, https://links.lww.com/PA9/A69. When analyzing the monthly incidence of TA over the study period, the majority of cases were found to be in the latter 6 months of the year (Fig. 3).

**Table 2.** Pearson’s correlation coefficients (*P* values) for each environmental parameter in Greater Sydney, Northern NSW, Southern NSW and all regions combined

	Greater Sydney	Northern NSW	Southern NSW	All regions combined
Rainfall (2005–2021)	−0.15 (0.64)	−0.46 (0.13)	−0.20 (0.53)	−0.13 (0.68)
Temperature (2005–2021)	0.26 (0.42)	−0.20 (0.53)	−0.27 (0.39)	−0.22 (0.48)
9 am and 3 pm dewpoint temperature (2005–2010)	−0.22 (0.49), −0.22 (0.50)	−0.42 (0.17), −0.46 (0.12)	−0.42 (0.18), −0.41 (0.18)	−0.27 (0.39), −0.27 (0.40)
% Area burned (2016–2021)	−0.94 (0.01)	−0.40 (0.51)	0.25 (0.69)	−0.94 (0.02)

NSW, New South Wales.

Overall, a greater area burnt was associated with a reduced number of TA presentations in NSW. TA cases appear to also be less frequent with more rainfall and higher dewpoint temperatures (more humidity) in NSW; however, for temperature, results were inconsistent across the 3 regions.

## 4. Discussion

Tick-induced allergies—TA and mammalian meat allergy after tick bite—have both been reported more frequently within the last 2 decades [18], and are most prevalent in Australia, particularly in the Northern Beaches region and within the Sydney Basin of NSW.

This study is the first to examine the relationship between environmental variables and the rates of TA in coastal NSW. While patient-related variables such as age, comorbidities, atopic status, and blood type have been investigated by others [19], tick survival to the adult form, the only life stage known to trigger TA [18, 20], may rely in part upon environmental variables and therefore impact the number of presentations of TA. Here, the recorded number of TA presentations is an indirect means of identifying the prevalence of adult ticks. While previous studies have examined tick distribution across NSW, climatic preferences of *Ix. holocyclus*, rates of animal-related tick paralysis with relation to seasonal weather, as well as survival of ticks in laboratory-controlled environments, there are no studies to date that examine the abundance of ticks in relation to the bushfire extent, rainfall, humidity, and temperature in their natural habitat [13, 21–24].

This study demonstrates that during bushfire seasons with large areas of land burned, there was a reduced number of TA presentations. The main reasons for this finding are likely 3-fold: destruction of tick habitat with high temperatures not only would have destroyed the vegetation that ticks rely on for shelter and from which to climb onto hosts [25, 26], but also may have led vulnerable tick eggs and larvae to desiccate in such dry conditions [13, 24]. Further, ticks are dependent on a large variety of native hosts, such as rodents, rabbits, birds, wallabies, koalas, possums, and foxes [14, 27–30]. It is possible that for any ticks that do survive bushfires, the reduced numbers of available hosts prevent adult ticks from becoming engorged, which in turn causes them to be more prone to desiccation, resulting in their death [13, 24]. Moreover, during these periods of increased environmental hazards, the public is likely to avoid exposure to bushfire-prone areas, thus the likelihood of tick bites and subsequent anaphylaxis is lowered. To better determine these postulated effects of bushfires on adult tick numbers, it may be useful to examine the number of TA cases in the months following bushfire seasons, because whilst the bushland regenerates and host animals return to their habitats, TA cases should still be low.

There was variability in the correlation between temperature and TA cases across Northern coastal NSW, Southern coastal NSW, and Greater Sydney; however, when taken together, reduced numbers of TA were seen with higher average monthly temperatures, which may reflect the tendency for adult ticks to desiccate in hotter conditions [13, 24]. It is unusual for the direction of the correlation to differ between regions, as the species of tick remains the same. It could be speculated that in different areas, specific geographical features, such as mountains or nearby bodies of water, form a microclimate for ticks that may not be reflected purely by measurements of weather variables. TA cases were also negatively correlated with higher rainfall and higher dewpoint temperatures in NSW, contrary to reports of improved survival of tick eggs and larvae in higher humidity [13, 24]. Previous studies of *Ix. holocyclus* and other tick species have indicated that higher rainfall and humidity, with lower temperatures, increase tick abundance; however, the results of this study do not reflect this [13, 24, 30, 31]. TA is caused by adult ticks, which are less vulnerable to temperature and humidity changes, whereas humidity promotes the survival of eggs and larvae [13, 24]. Therefore, the results of this study may reflect the adult stage of the life cycle that the ticks were predominantly in during the periods of higher TA cases. Furthermore, higher rainfall seen in flood seasons may have destroyed tick habitats and reduced tick host availability, thus reducing tick abundance [32]. Alternatively, higher rainfall may also lead to reduced outdoor human activity and consequently reduced likelihood of tick bites and tick anaphylaxis. Ultimately, these findings should be interpreted carefully, as many data points did not reach statistical significance. Consideration of both tick and human factors is also required when analyzing such associations.

In this study, the majority of TA cases presented in the second half of the year, which is consistent with previous research demonstrating that *Ix. holocyclus* adults, which are the life stage largely responsible for TA, predominate in the warmer months of spring [12]. Because the association between tick abundance and individual environmental variables is currently inconsistent with previous studies, public education on tick prevention strategies may be more effective if disseminated with regard to seasons instead of environmental conditions.

### 4.1. Limitations

There are a few limitations relating to the use of a large, single-source patient database. For one, the retrospective collection of data is subject to recall bias as patients were followed up in the clinic after their initial ED presentation. The database is also incomplete as information was retrieved from the patient record, and not all details were asked at the time of review. Moreover, a large number of these patients are located in the Northern Beaches and North Shore area of Metropolitan Sydney. While it is possible that the distribution of data towards the Northern Beaches area merely reflects the ideal environment for *Ix. holocyclus* to thrive in, it is more likely due to these patients being seen by a consultant located in this area. Furthermore, the addresses provided are the patients’ residences, not necessarily where they were bitten. Thus, the low power data makes it difficult to form significant correlations with weather variables in regional NSW.

The present study is limited by taking averages of environmental variables over several years rather than analyzing each year individually, to account for occasional gaps in data and small sample sizes. It would be useful to consider yearly fluctuations in TA cases had the sample size been larger, as this would provide insight into whether seasonal patterns in tick abundance are consistent each year. Despite this approach providing a better general overview of the relationships between environmental data and TA incidence, it is also important to consider seasonal interactions, as tick larvae have been shown to predominate during autumn, nymphs in winter and adult ticks in spring, and more humid summers are positively associated with the survival of eggs and engorged females [30, 33].

### 4.2. Future directions

Further studies should continue characterizing the environmental preferences of ticks by studying the patterns of tick-induced allergies in relation to the weather, particularly in areas outside of the Northern Beaches. By understanding the effects of environmental variables on TA cases and the environmental conditions that cause increases in tick abundance, a basis for tick season predictions may be formed. Forecasting incoming tick seasons may then allow hospitals to prepare for an increased number of TA presentations by ensuring training is updated and resources are available. Forecasts may also provide a basis for future public health initiatives to educate the public about tick bite prevention during or preceding tick seasons. Over time, this information may even be used in the context of global climate change to contribute to predictions of future distributions of ticks and the incidence of tick-borne allergies and diseases [10, 11, 34]. Furthermore, this study was limited to environmental variables and disregarded the effects of host animals as part of the tick habitat, despite various other studies demonstrating the impact of host animals on tick survival [28–30]. Further studies may look at the relationship between host animal abundance and TA cases, for example, by looking at the timing of fox baiting in local council areas and its relationship to TA presentations.

## 5. Conclusion

TA cases tend to fall when bushfire extent is greater and possibly when temperatures, humidity, and rainfall are higher. However, due to conflicting findings with previous studies and variation across different regions of NSW, the influence of temperature, humidity, and rainfall needs to be further investigated with more cases outside of Greater Sydney. This study has highlighted the complex interaction between ticks, their hosts, their habitat, and the weather, which is yet to be fully understood but is vital for future public health initiatives to prevent tick bites and TA.

## Conflicts of interest

The authors have no financial conflicts of interest.

## Authors contributions

This project was conceptualized by SvN, AR and MB. MB organized SvN’s patients into a database. Environmental data was collected and organized by KD. DB provided recommendations on statistical analysis and presentation. KD reviewed the literature and prepared the initial manuscript draft, with all authors providing feedback and contribution for the final draft.

## Supplementary Material
