# Supplementary material for: Environmental variables influencing tick anaphylaxis presentations: An observational study
Source: Asia Pac Allergy. 2025 Oct 6;16(2):85–91. doi: 10.5415/apallergy.0000000000000222 (PMC13060779; doi:10.5415/apallergy.0000000000000222)
Supplement: Supplementary file 1 [file pa9-16-085-s001.pdf]

APPENDICES

Appendix 1 - Weather stations selected for data collection

NSW region	Local government area	Weather station
**North Coast**	Tweed	Murwillumbah (Bray Park)
	Byron	Byron Bay (Cape Byron AWS)
	Lismore	Lismore Airport AWS
	Ballina	Ballina Airport AWS
	Richmond Valley	Casino Airport AWS
	Kyogle	Wiangaree Post Office
	Clarence Valley	Yamba Pilot Station
	Coffs Harbour	Coramba (Glenfiddich)
	Bellingen	Dorrigo (Old Coramba Rd)
	Nambucca	Bowraville (Cow County)
	Kempsey	Kempsey Airport AWS
	Port Macquarie-Hastings	Yarras (Mount Seaview)
**Hunter**	Mid-coast	Taree Airport AWS
	Upper Hunter	Scone Airport AWS
	Dungog	Paterson (Tocal AWS)
	Newcastle	Newcastle Nobbys Signal Station AWS
	Muswellbrook	Muswellbrook (Lindisfarne)
	Singleton	Elderslie, Singleton Defence AWS

	Maitland	Maitland Belmore Bridge (Hunter River), Maitland Airport AWS
	Port Stephens	Nelson Bay (Nelson Head), Williamtown RAAF
	Cessnock	Cessnock Airport AWS
	Lake Macquarie	Eraring (Payten St), Cooranbong (Lake Macquarie AWS)
**Central Coast**	Norah Head AWS<br>Mangrove Mountain AWS	
**Metropolitan/Greater Sydney**	Sydney	Canterbury Racecourse AWS
	Southeastern Sydney	Sydney Airport AMO
	Southwestern Sydney	Bankstown Airport AWS
	Northern Sydney	Collaroy (Long Reef Golf Club), Terrey Hills AWS
	Western Sydney	Parramatta North (Masons Drive)
	Nepean Blue Mountains	Mount Boyce AWS, Katoomba (Farnells Rd)
**Southern NSW**	Hilltops	Young Airport
	Upper Lachlan	Taralga Post Office
	Wingecarribee	Moss Vale AWS, Bowral (Parry Drive)

	Yass Valley	Burrinjuck Dam
	Goulburn/Mulwaree	Goulburn Airport AWS
	Queanbeyan-Palerang regional	Braidwood Racecourse AWS
	Eurobodalla	Moruya Airport AWS
	Snowy Monaro regional	Cooma (Kiaora), Cooma Visitors Centre, Cooma Airport AWS
	Bega Valley	Bega AWS
**Illawarra**	Wollongong	Bellambi AWS
	Shellharbour	Albion Park (Shellharbour Airport)
	Kiama	Jamberoo (the Ridge), Kiama (Bombo Headland)
	Shoalhaven	Nowra Ran Air Station AWS

AWS: automatic weather station

Appendix 2. Scatter plots showing the correlation between weather data and tick anaphylaxis cases in Northern coastal NSW and Southern coastal NSW.

[insert appendix 2 file]

Appendix 3. Map of entire study region (constructed with Google MyMaps)

[insert appendix 3 image file]
