# Supplementary material for: Environmental variables influencing tick anaphylaxis presentations: An observational study
Source: Asia Pac Allergy. 2025 Oct 6;16(2):85–91. doi: 10.5415/apallergy.0000000000000222 (PMC13060779; doi:10.5415/apallergy.0000000000000222)
Supplement: Supplementary file 2 [file pa9-16-085-s002.pdf]

Appendix 2 – scatter plots for Northern and Southern coastal NSW regions

**A**

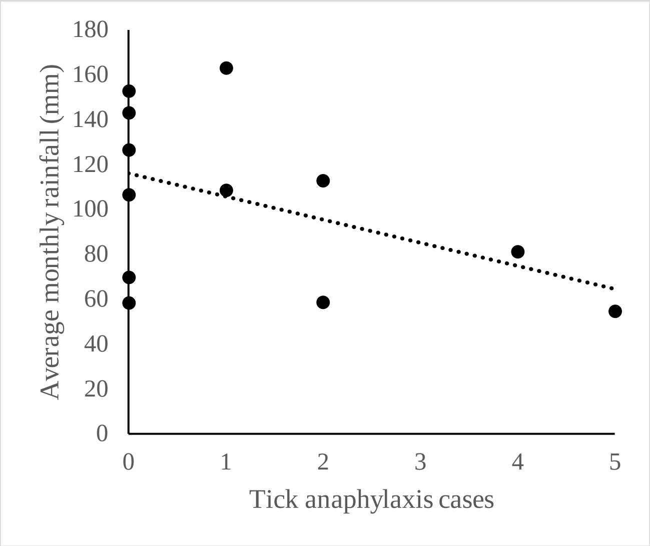


**B**

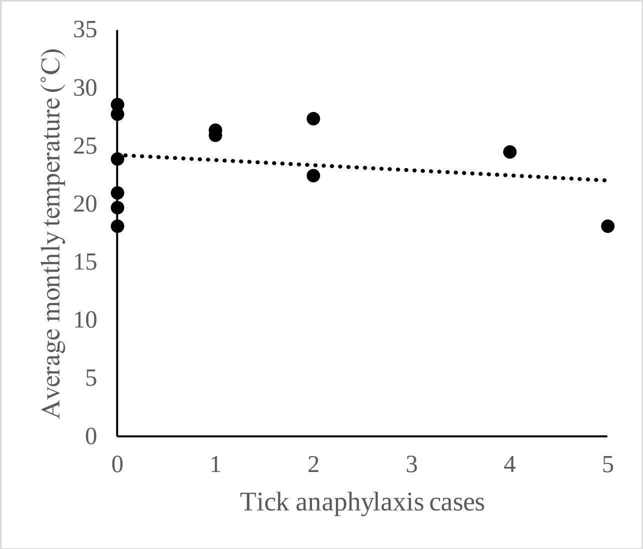


**C**

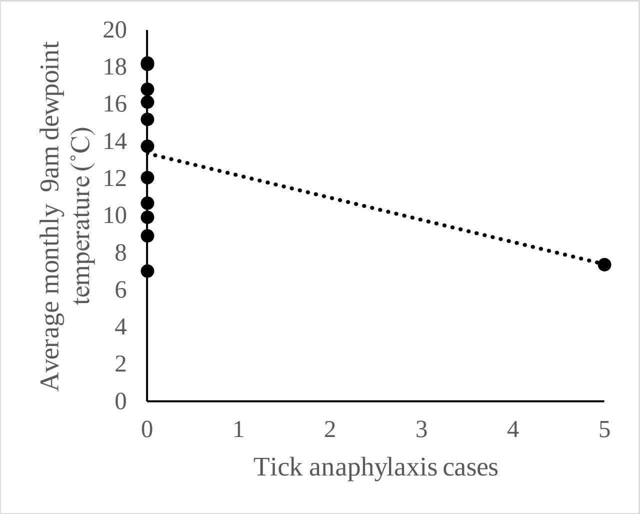


**D**

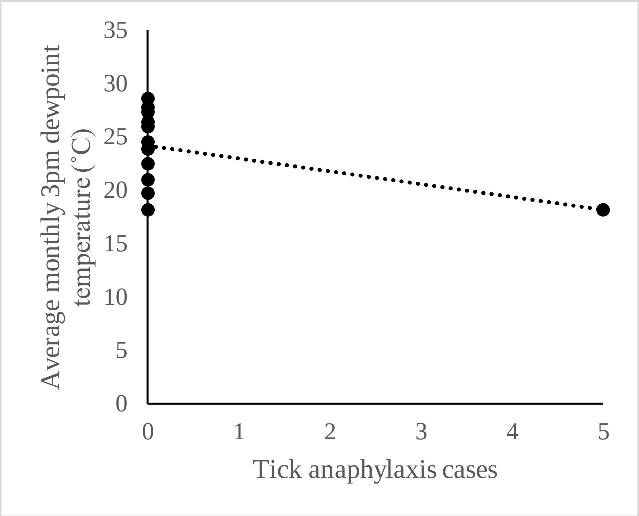


**E**

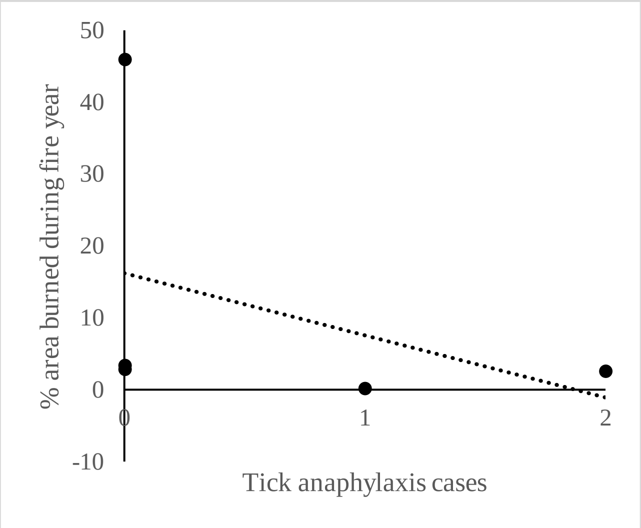


A: Northern NSW - Rainfall vs Cases 2005-2021

B: Northern NSW - Temperature vs Cases 2005-2021

C: Northern NSW - 9am Dewpoint vs Cases 2005-2010

D: Northern NSW - 3pm Dewpoint vs Cases 2005-2010

E: Northern NSW - % area burned vs Cases 2016-2021

Appendix 2 – scatter plots for Northern and Southern coastal NSW regions

**F**

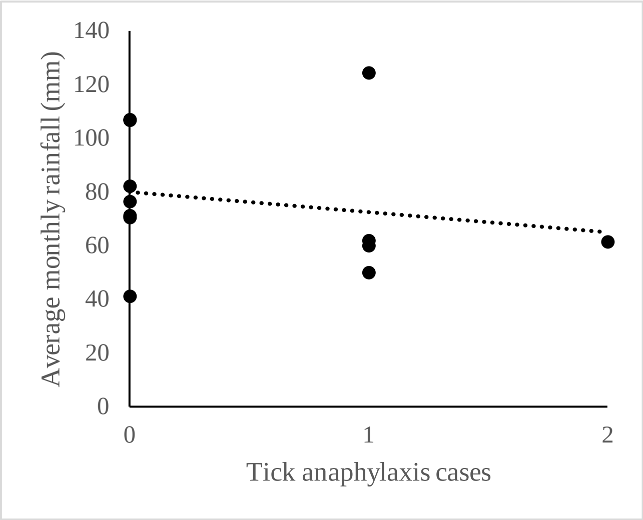


**G**

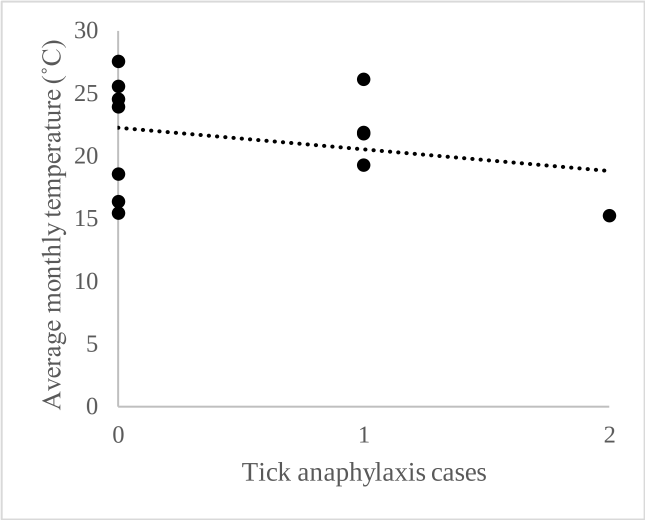


**H**

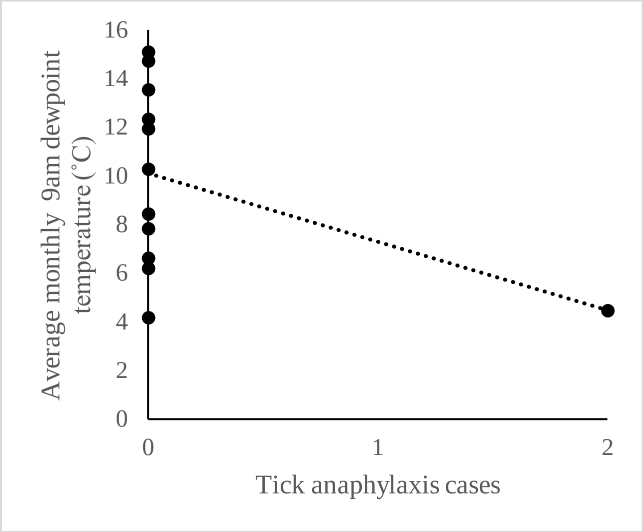


**I**

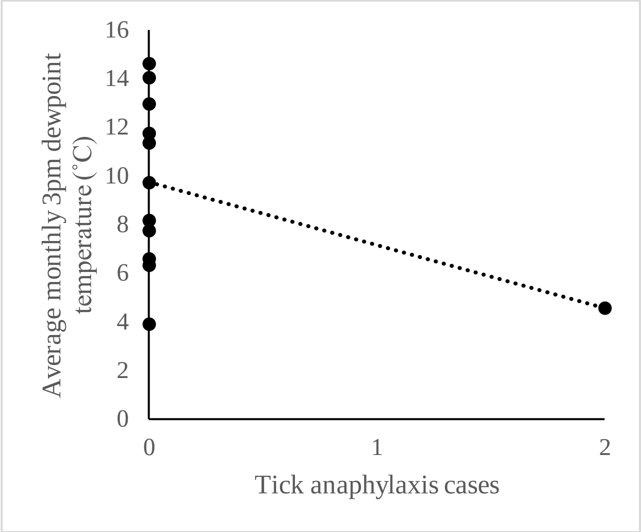


**J**

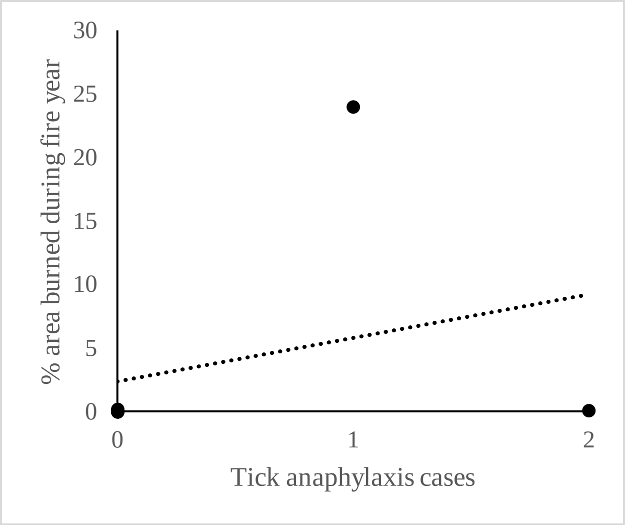


F: Southern NSW - Rainfall vs Cases 2005-2021

G: Southern NSW - Temperature vs Cases 2005-2021

H: Southern NSW - 9am Dewpoint vs Cases 2005-2010

I: Southern NSW - 3pm Dewpoint vs Cases 2005-2010

J: Southern NSW - % area burned vs cases 2016-2020
