# Supplementary figures and images for: Environmental variables influencing tick anaphylaxis presentations: An observational study
Source: Asia Pac Allergy. 2025 Oct 6;16(2):85–91. doi: 10.5415/apallergy.0000000000000222 (PMC13060779; doi:10.5415/apallergy.0000000000000222)

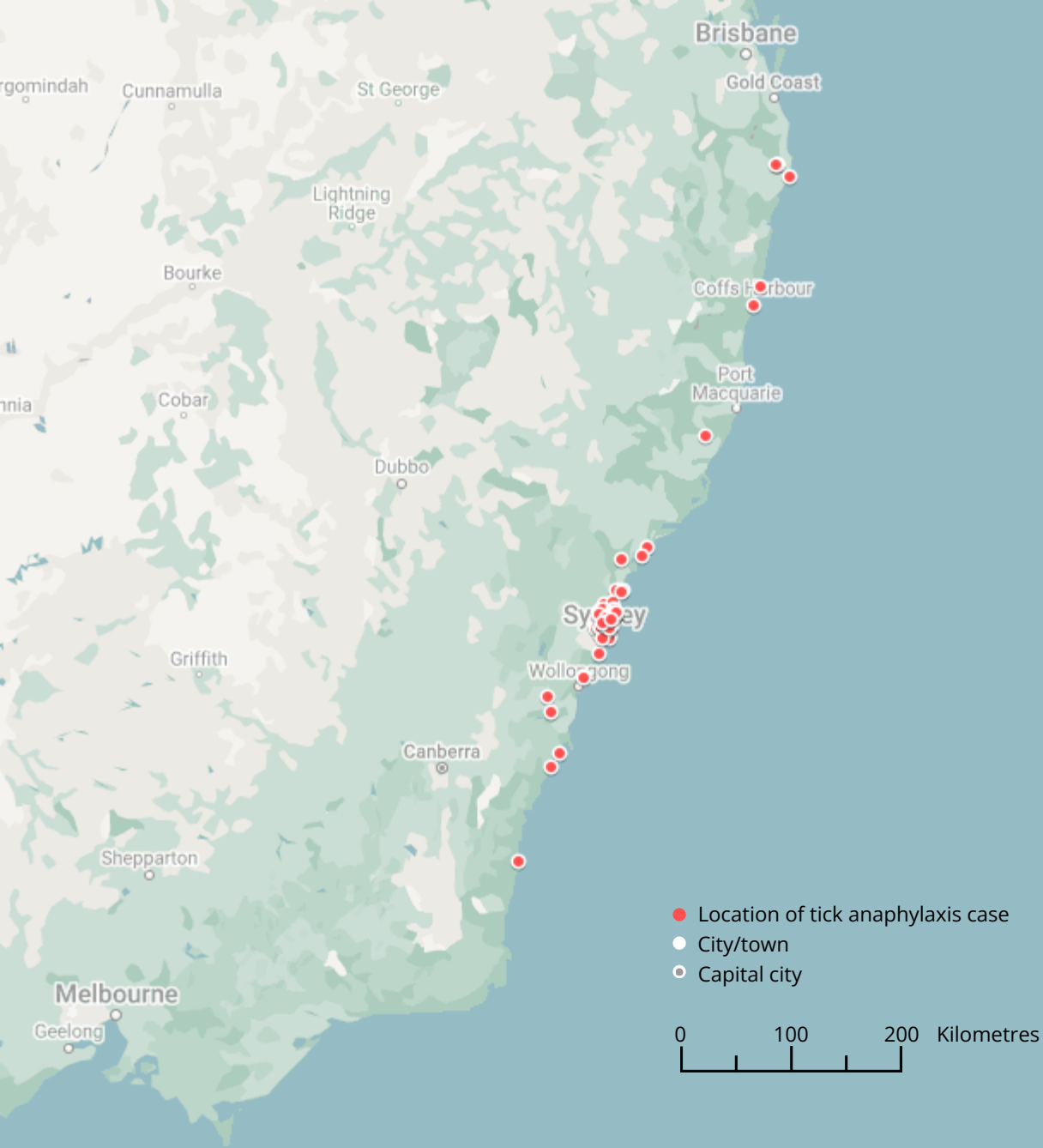

Supplement: Supplementary file 3 [file pa9-16-085-s003.pdf]
